# A call for culture-centred care: exploring health workers' perspectives of positive care experiences and culturally responsive care provision to Aboriginal women and their infants in mainstream health in South Australia

**DOI:** 10.1186/s12961-022-00936-w

**Published:** 2022-12-12

**Authors:** Nina Sivertsen, Janiene Deverix, Carolyn Gregoric, Julian Grant

**Affiliations:** 1 Caring Futures Institute, College of Nursing and Health Sciences, Flinders University, GPO Box 2100, Adelaide, SA 5001 Australia; 2 Department of Health and Care Sciences, UiT Arctic University of Norway, Campus Hammerfest/Kautokeino, Tromsø, Norway; 3 Child and Family Health Service, Women’s Children’s Health Network, 295 South Terrace, Adelaide, SA 5000 Australia; 4 School of Nursing, Midwifery and Indigenous Health, Charles Sturt University, Panorama Ave, Bathurst, NSW 2795 Australia

**Keywords:** Continuity of care, Aboriginal, Maternal–child health, Prenatal care, Antenatal care, Infant, First nations health, Health systems, Health services

## Abstract

**Background:**

Aboriginal women and their infants experience significant disadvantage in health outcomes compared to their non-Aboriginal counterparts. Access to timely, effective, and appropriate maternal and child health care can contribute to reducing these existing health disparities. This research sought to explore factors that contribute to continuity of care for Aboriginal women and their infants living in metropolitan South Australia. This paper reports on the perspectives of health care workers in mainstream health services from the antenatal period to the end of an infants’ second birthday. It explores health workers’ perspectives of what contributes to positive care experiences and satisfaction with care provided to Aboriginal women and their infants in mainstream health.

**Methods:**

Eight focus groups were held with 52 health professionals. Participants included Aboriginal Cultural Child and Family Support Consultants (*n* = 7), Aboriginal Maternal Infant Care Workers (*n* = 3), Midwives (*n* = 3) and Child and Family Nurses (*n* = 39). Data was inductively coded and thematically analysed.

**Results:**

Three key themes emerged: the system takes priority, culture is not central in approaches to care, and ‘we’ve got to be allowed to do it in a different way’.

**Conclusions:**

This research highlights a lack of continuity of care for Aboriginal families accessing mainstream health services from the antenatal period through to an infants’ first 1000 days of life. This research has implications for communities, and it calls for strategies to enhance continuity, and healthcare services to provide appropriate and culturally safe care. Findings will inform and guide future changes to improve continuity of care for Aboriginal families and infants in the first 1000 days.

**Supplementary Information:**

The online version contains supplementary material available at 10.1186/s12961-022-00936-w.

## Background

Aboriginal Australians experience significant disadvantage in health, life expectancy, education, employment and living standards compared to other Australians. This is further compounded by inadequate service provision including insufficient service delivery, limited staffing, and culturally unsafe practices [1]. Health inequities begin before birth and continue throughout the life cycle [2]. Reducing disparities from the very beginning of life is therefore vital. Of particular concern, the maternal mortality rate of Aboriginal women is nearly three times higher than non-Aboriginal women [3], with Aboriginal women having higher rates of gestational diabetes and smoking during pregnancy [4]. Aboriginal babies are nearly twice as likely to be born preterm and have low birth weight. The perinatal mortality rate of Aboriginal infants is double those of non-Aboriginal infants [1, 5–18].

The complexity of factors that contribute to these disparities in health care for Aboriginal women and children include lack of culturally appropriate health services, institutional racism, lower educational attainment, poverty and the ongoing effects of colonisation [7, 10, 13, 19]. Remoteness adds additional challenges to health service provision and delivery thereby further decreasing Aboriginal maternal and infant health outcomes [1]. It is no surprise then that Aboriginal women are less likely to engage in mainstream antenatal care than non-Aboriginal women [20]. Fear and anxiety, from not feeling culturally safe, means that Aboriginal women access fewer maternal and infant health care services than non-Aboriginal women [21, 22].

Engagement is increased when there are ongoing relationships with health workers, especially those who are also of Aboriginal descent [20]. In Australia and internationally the most successful models of midwifery care for Indigenous women are those that support relationship development through continuity (see for example [12, 15]). Continuity of care refers to service models in midwifery practice that integrate continuity of services and/or continuity of carer during antenatal, labour, birthing and beyond to post-natal care of infants [23]. Such care is considered more culturally safe than other models and can lead to more families engaging in perinatal health care [24]. Yet continuity for Aboriginal families is limited by a western biomedical approach to care, structural constraints to healthcare provision such as limited resourcing and unyielding policies, and the approach and attitudes of clinicians [25].

Providing care that is culturally safe is a core requirement of nursing and midwifery practice [26]. Despite this, it is currently not consistently demonstrated across the first 1000 days of Aboriginal infants’ lives [25]. Indeed, Midwives have limited knowledge about Aboriginal women’s cultural needs and limited access to cultural education [27]. South Australian child and family health Nurses had little understanding of what constitutes racist practice and inconsistencies in knowledge of the impact of racism on children and families [28]. Moving from individual practice to models of care, there is very limited research exploring how continuity of care is enacted by Midwives and Nurses with Aboriginal families transitioning from antenatal through to child and family health care across the entirety of first 1000 days [25]. Further, there is little evidence marking the contribution made by Aboriginal workers to the health care team. It is not known how continuity is enacted within and between mainstream health services for an infant’s first 1000 days. Also, it is not known how and if continuity of care is experienced as culturally safe.

## Methods

### Aims of the study

The overall purpose of this study was to [1] explore and identify health workers’ capacity to provide continuity of care that is thought to be experienced as culturally safe by Aboriginal families with infants in the first 1000 days of life, and [2] to explore with Aboriginal family experiences of care as continuous during the first 1000 days of their infants’ life. This paper reports on data related to aim [1] provided by Child and Family Health Nurses (CaFHNs), Midwives, Aboriginal Maternal Infant Care (AMIC) workers and Aboriginal Cultural Child and Family Support Consultants (ACCFSCs) about their perceptions and experiences of care provision.

### Research design

This qualitative study was conducted in collaboration with a state-wide mainstream health service responsible for providing care to Aboriginal families across the first 1000 days in Adelaide, South Australia. Ethical approval for this research was granted by the Flinders Social and Behavioural Research Ethics Committee Project Number OH-00185, the Aboriginal Health Research Ethics Committee project number 04-18-769, and the Women and Children’s Health Research Ethics Committee project number HREC/18/WCHN/90.

### Participants and recruitment

Practitioners working as ACCFSCs, AMIC Workers, Midwives or CaFHNs in seven metropolitan service areas and one rural service area, were invited to participate in the study. In general, CaFHNs and Midwives work with women of any cultural background, whereas AMIC and ACCFSCs work only with Aboriginal women, or women giving birth to Aboriginal babies. Via an initial convenience sample, then a purposive sampling [29] technique, participants were recruited via email through study sites in clinical and community settings working with Aboriginal families.

The participant and recruitment context is made up of a large portfolio of health networks and services to people in South Australia [30]. The Aboriginal Family Birthing Program provides antenatal and postnatal care in a culturally sensitive environment, with the support of Midwives, doctors, AMIC workers, social workers, and family support workers [31]. Following the birth of a baby, families in South Australia are offered a consultation with the Child and Family Health Service (CaFHS), who supports families in South Australia with health and development checks of children aged 0–5 years. The Child and Family Health Service (CaFHS) has a number of Aboriginal Cultural Child and Family Support Consultants (ACCFSCs), CaFHNs and Aboriginal clinical leaders whose role is to support parents and carers of Aboriginal identified infants [32]. Overall, there were 18,574 registered births in South Australia in 2020, where of 1025 were Aboriginal and Torres Strait Islander births [33].

### Data collection and analysis

In total, eight focus groups (FG) were held with 52 health and community professionals. The shortest interview was 25 min and the longest 1 h and 3 min and were guided by a semi-structured interview schedule.

FGs were offered with homogenous groups to reduce potential issues of unequal relations of power between the disciplines. This strategy is shown to yield clearer, more valid and generalisable results [34]. They also enabled interactions between the participating health care professionals to gather broad range of views and experiences [35–37]. A FG guide was used consistently across all FGs (available in Additional file 1).

Participants included ACCFSCs (FG = 1, *n* = 7), AMIC Workers (FG = 1, *n* = 3), Midwives (FG = 1, *n* = 3) and Child and Family Nurses (FG = 5, *n* = 39). The sample included participants who identified as Aboriginal and those who did not (see Table 1). Participants were provided with an information sheet and written consent was obtained prior to the interview. Health and community professionals’ experience ranged from 1–45 years. During the focus group, participants were asked about continuity of care, management of transitions between services and cultural safety.

**Table 1:** Participants

Health professional group	Number of FGs	Number of participants	Participants identifying as Aboriginal	Participants identifying as non-Aboriginal participants	Work experience in years	
					Min	Max
ACCFSCs	1	7	7	0	1	3
AMIC workers	1	3	3	0	8	10
Midwives	1	3	1	2	9	30
CaFHNss	5	39	0	39	1	45

### Analysis

Audio recordings from focus groups were transcribed for analysis and coding, which was undertaken as a team with multiple authors present through all stages. Data was firstly inductively coded using NVivo™ software [38] for content and meaning, then thematically analysed by the research team guided by Braun and Clarke’s [39] thematic analysis framework.

Initial descriptive coding consisted of reading through qualitative data and coding passages that provided answers to the research questions. These were clustered according to emerging patterns. An interpretive second cycle involved filtering, highlighting, and refining the meanings of codes into categories and concepts, then building analytical themes [40]. During this stage of iterative team discussion and synthesis, particular care was taken to view the findings through a theoretical lens of cultural safety [41].

In the first stage descriptive analysis, participants identified that they did not feel enabled to provide continuity of care. Poor continuity of care was observed and experienced in practice with participants reporting inconsistencies in transitions between services. The majority of participants (*n* = 48) believed that the lack of continuity of care resulted in culturally unsafe practices, and those individuals that did feel as though they could provide culturally safe care, were doing it despite the system. They identified prioritising relationships within and between systems, including amongst co-workers and with consumers, clients, and patients. These staff identified working beyond standard practice within the environment of the system.

Findings are presented by weaving text and quotations; selected spoken words illustrating participant meaning are blended with narrative text as evidence, explanation, and illustration to deepen understanding, give participants a voice, and enhance readability. There is clear distinction between the author’s narrative and the verbatim quotations, which are written in quotation marks and italicised [42].

In the second stage data were explicitly interpreted through a lens of cultural safety. Ramsden [43], a Maori Nurse leader in New Zealand, developed the concept of cultural safety due to health inequities resulting from colonial health care systems, and advocated for change to service delivery. Historically, these processes disregarded the illness and health belief systems of the Maori, and instead, privileged those of the dominant 'white' culture in the construction of the healthcare system [44]. Cultural safety reminds us all in health care to reflect upon the ways in which our policies, research and practices may continue to inflict traumas upon Aboriginal people [44]. In this research we look *through* cultural safety as an interpretive lens to interrogate ways in which health policies, systems and service delivery is perpetuating systemic colonisation. The analysis instead focusses on systemic reflection and how we can use cultural safety principles to discuss equity, restorative policies, and justice, negotiated partnership [45] and two-eyed seeing to decolonise care and service delivery [46].

**Fig. 1:**
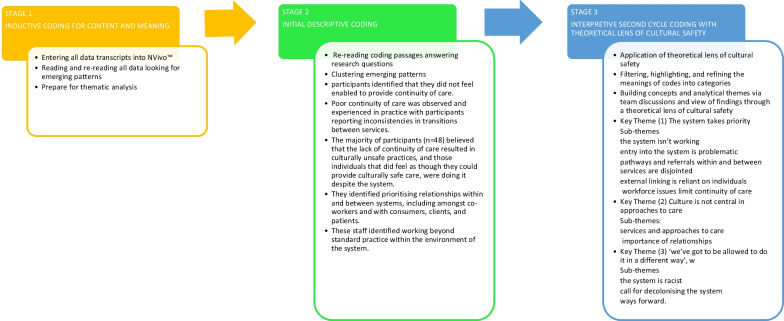
Analysis and key themes

This interpretive process resulted in categorisation into three main themes (see Fig. 1).

## Results

Three key themes were identified in the data: (1) The system takes priority, with sub-themes of the system isn’t working, entry into the system is problematic, pathways and referrals within and between services are disjointed, external linking is reliant on individuals, and workforce issues limit continuity of care; (2) culture is not central in approaches to care, with sub-themes of services and approaches to care, and importance of relationships; and (3) ‘we’ve got to be allowed to do it in a different way’, with sub-themes of the system is racist, call for decolonising the system, and ways forward.

### Main theme 1 the system takes priority

#### The system isn’t working

Discussions identified that the family journey is not supported by the system pathway and the system appears not to have continuity at the core. *“There are just too many different things out there and there are just too many gaps to fall through, and the people right up the top, they need to really work together to get it right”.* (FG4 CaFHNs). The system supporting Aboriginal families is reported to be fragmented with interactions between services are often disjointed and without integration.

*“I think that’s the problem, though, of not having the continuity. Knowing that you’ve got the continuity at the triage [start of care journey antenatally], because if you knew you had the continuity at the triage, you could follow up”* (FG2 Midwives). Discussion in FG 2 identified the frustration of not always knowing how to support families in the system because continuity was not prioritised depending on the models of care presented to a family and how they were supported to make choices. They noted that while attempts had been made to address this, they were not always successful. *“If they opt for our kind of care in our [mainstream] service then we’ll try for continuity with an AMIC worker provided but that doesn’t always [happen]”* (FG2 Midwives).

Within the current system there is service repetition with multiple services doing exactly the same thing*. “We had a lady just recently that’s got multiple services in there that we had no idea that we were doing. So, continuity of care goes out the window then because you’ve got multiple services that aren’t talking to them, and I think that a lot of problems with a lot of these clients [Aboriginal families] is because their services aren’t talking”* (FG4 CaFHNs). With a lack of communication between services, different services may not be aware of all the support a family is receiving.

#### Entry into the system is problematic

Continuity can be further challenged when families are seeing multiple services and different workers within services. Although there is a state-wide pregnancy information phone line (pregnancy related), approaches to entering the system are not consistent with different entry points and varying information about services available. Participants in FG 1 argued that Aboriginal families may not be informed of all their options and services available. *“The mums are not told in the hospital that they might be able to go to a local Aboriginal health service …they don’t get that opportunity. If it means that they want to go down that path, then a track needs to be developed, a pathway needs to be developed so that that can happen”* (FG1 CaFHNs). Participants in FG 2 identified that taking particular antenatal pathways excludes Aboriginal families from other services. For example, intake systems do not align. *“Their intake system doesn’t necessarily match with how we operate so there’s a gap”* (FG2 Midwives). If families want to enrol in the mainstream birthing program, they are excluded from the Aboriginal health service (birthing program). A major challenge identified in this group was for health workers and families to know about all the different services and programs.

A further concern was that the mainstream state-wide system enforced limits on the total number of women assisted by culturally appropriate services. This meant that if Aboriginal women did not enrol early enough in their service of choice, they missed out. Additionally, Aboriginal women could not have both a mainstream midwife and Aboriginal care. For example, the MGP (Midwifery Group Practice: enabling women to be cared for by a primary midwife) is popular and sought out early by informed women. *“The other thing too with the continuity models generally, I’m thinking about MGP as well, they’re very popular. And a lot of the well-educated women will be seeking them out early. So, those places fill very quickly”* (FG2 Midwives). Women who do not get into MGP may then miss out on continuity with the Aboriginal Family Birthing Program because they are then in a later stage of pregnancy. Further, restrictions occur at a system level when for example, the location of services, funding requirements that family enrol antenatally and follow through, families may not be informed that a service is voluntary, and restrictions on how care is provided.

#### Pathways and referrals within and between services are disjointed

Post-birth pathways for all families, including Aboriginal families, begin with CaFHS (Child and Family Health Service). Entry into this service pathway is not through personal contact but is automated with predetermined service progression. The CaFHS Nurses provide a follow-on service from Midwives’ home visits. This is problematic for Aboriginal families as AMIC workers are excluded from the formal pathway. *“It would be good for those forms, if the women are Indigenous or baby is Indigenous, that those forms don’t just go to a Nurse, that they are automatically sent to the ACCFSCs for the ACCFSCs to be the primary carer”* (FG3 ACCFSCs).

Information technology systems to support continuity of care and referral between services within the state-wide mainstream system were reported to be lacking. Participants expressed frustrations with a system that relies on old technology, paper forms and fax, to connect community-based child and family health service (CaFHS) with families after giving birth. *“That piece of paper referral has got to follow that mum all the way through”* (FG1 CaFHNs). These apprehensions progressed to concerns about lack of continuity for the family across the entire pathway from antenatal care to birthing and through to community-based care with child health services. For example, understandings of predetermined generic pathway into CaFHS, where hospitals do not additionally (or alternately) send referral forms onto Aboriginal health services. *“It never goes to the Aboriginal Health Service so they can go out”* (FG4 CaFHNs). As a consequence of not referring to Aboriginal services and workers *“many of our clinic Aboriginal families might not get that option of having a worker. So, I think that's a gap”* (FG8 CaFHNs).

Within the mainstream network discussion from FG 5 identified that the system appeared to be a series of services that were not integrated. *“It's very separated. And its forced separation so I think we're seen as different people and services … the ACCFSCs service and your service [Aboriginal Maternal Infant Care] are just very different, and they operate separately”* (FG5 AMIC). Aboriginal families can be assisted by discrete services within the same system rather than experience interconnected services.

#### External linking is reliant on individuals

Referral pathways between external services were reported as largely reliant on individual knowledge and connections. For example, women were not informed by mainstream services of Aboriginal specific services and Midwives were unsure of referral processes and services offered by other organisations. Referrals and transitions between services were often up to individuals and how they work, or not, with other services. Care was reported as being fragmented from the beginning. *“The first 20 weeks. It’s already fragmented, she’s got three different types of care going on”* (FG2 Midwives).

Some services were reluctant to let go of clients and refer on. *“I think sometimes we just think, we’re just going to hang onto this client now, and even sometimes down the track things sort of fester up and there are other issues around the families that we’re like, oh, hell, we haven’t got the services. So, it’s linking the families and letting go, as well, to a service that is better suited for that family”* (FG4 CaFHNs). Rather than working collaboratively, they may see others as competition. *“It’s always been kind of them against us sort of, but we we’re wanting the same outcome for the client”* (FG1 CaFHNs). This may be because services are concerned about their funding and jobs. *“It’s [organisation] become very protective and this surrounds people’s roles from there. I don’t know if it’s attached to funding, or what the motivator is”* (FG2 Midwives).

Processes for referral to interservice procedures are not well documented or known meaning that connection is often ad hoc. *“They’re informal links, there’s no roadmap for that connection”* (FG4 CaFHNs). The process is reliant on individuals to proactively know and connect with other services. As a participant from FG 7 reflected *“All of them [other agencies] have got different arms into homelessness, family supports, and I need to navigate that system to work out who would be the best”* (FG7 CaFHNs). Some families may need a little more support to encourage them to access other services. *“Pathways exist for everybody, but sometimes some families do need a little bit more support in actually encouraging to access services that are quite scary for anybody”* (FG4 CaFHNs). Within the mainstream health service processes exist to support this and when there are enough staff this appears to work well. *“We have an arrangement for those families who don't want to engage with us and have had bad experiences in the past, what's it called; a warm handover, which is joint visit”* (FG8 CaFHNs).

#### Workforce issues limit continuity of care

Professional scopes of practice limited what health workers can do. For example, Midwives provide care across the antenatal, intrapartum, and postnatal period. AMIC workers reported antenatal contact but being unable to support women during the intrapartum period. In another example, ACCFSCs working in CaFHS identified that they could take a more active clinical role for Aboriginal families during visits. *“The ACCFSCs should be the primary carer; they should be the lead. But it’s not, it’s Nurse led”* (FG3 ACCFSCs).

The current workforce has insufficient Aboriginal workers. *“We've got one Aboriginal cultural consultant, one. It's way too few to actually give them [recipients of care] the care that they need culturally”* (FG6 CaFHNs). This means that non-Aboriginal Nurses often visit Aboriginal families unaccompanied by an Aboriginal worker. A CAFHS Nurse (FG8) stated *“I haven't done many [visits] with an ACCFSCs because there hasn't been the availability of ACCFSCs … I would have liked the ACC because I don't feel that I can provide them with a smidgeon of what they could be”* (FG8 CaFHNs). At times this workforce crisis meant that ACCFSCs were placed in the awkward position of representing a family that they did not know during urgent case reviews. In these situations, they felt the representation tokenistic as they could not effectively undertake this role.

Although many individual workers were supportive of continuity of care in principle and tried to enable this through their approaches to care, the services they were able to offer made this difficult. *“We are trying with what resources we've got to offer …a good service but it's not the best service, it's the best we can do with what we've got at the moment”* (FG8 CaFHNs). For example, continuity was more difficult when families did not attend appointments. *“It’s difficult to provide services because they may just not turn up for whatever reason. There are lots of complexities in everybody’s lives”* (FG4 CaFHNs). Families *“might engage for a short time but then they would go walkabout. They would literally go… You couldn't get hold of them; you couldn't find them”* (FG6 CaFHNs).

### Main theme 2 Culture is not central in approaches to care

#### Services and approaches to care

Effective approaches to care need to be family and community centred [47]. From a policy and service perspective, mainstream health professionals are guided by a person-centred approach to care. *“It’s very individualised to what each woman needs to help her succeed and have good antenatal care”* (FG2 Midwives). Yet Aboriginal culture is community centred. Participants questioned whether, from an Aboriginal perspective, the person-centred model of care is appropriate. Whereas Aboriginal community-controlled services are run with Aboriginal contexts in mind, mainstream services were seeking to accommodate Aboriginal families but not being set up with their values in mind. Thus, with this mismatch, approaches to care can vary. Although there is individualised care and support, this relies on workers being proactive.

When care is Aboriginal led there is some continuity. In the perinatal space an AMIC worker explained *“We’re the primary, so we’re the consistent in our women’s journeys and then whatever midwife is available will come in”* (FG5 AMIC). However, while care could be somewhat smooth, this could also lead to some duplication. *“Sometimes it's the AMIC worker who has the relationship with the family. So, it's doubling up, we [the Midwives] need to find out what the AMIC worker is doing”* (FG2 Midwives). This may also be related to a ‘them and us’ attitude between health professionals with different levels of qualifications. *“We still find that the hierarchy in health is quite a barrier. That notion that the professional knows best is a significant barrier”* (FG3 ACCFSCs).

Respect and trust need to underpin care (Nursing and Midwifery Board of Australia, 2018). The values underpinning individual health professionals’ care of Aboriginal families largely support continuity of care. These include acknowledging Aboriginal ways of knowing and being, respecting individuality and choice, and developing trust. Aboriginal ways of knowing and being were acknowledged and prioritised by non-Aboriginal health professionals working with Aboriginal families and colleagues. *“We have to work alongside our different cultures, but it needs to be driven by them”* (FG7 CaFHNs). Similarly, *“I think co-working, about respectful relationships and understanding the importance of the role of Aboriginal [health workers]”* (FG3 ACCFSCs).

Respect for choice was valued. For example, recognising that some families may prefer to use more Aboriginal specific services than others. *“Sometimes the hurt and the past and the history is too deep to want to actually engage with us”* (FG6 CaFHNs). Alternatively, recognising there are families that do not want any support. *“In the big picture too is that some families don’t want anything. All they want is just to get on with their business, and they don’t want anybody to be around”* (FG4 CaFHNs). Problematically, non-Aboriginal health professionals’ perceptions of continuity of care may not be what families need. *“Our perception of continuity of care might not be what that family chooses to think of as needing. It’s a partnership that we need to have with them”* (FG7 CaFHNs).

Trust was a value shared by health professionals and they actively sought to develop this with families. However, Aboriginal families may not trust health care workers if they know too much about them beforehand and do not take time to get to know them. Different workers from the same organisation visiting families does not develop trust. Trust takes time to develop at the community and personal level. *“The community do get to know staff, it’s a lot of trust, and that’s invaluable when working with families who have got a lot going on”* (FG4 CaFHNs). It was reported that the CaFHS system is more focused on paperwork than people which impacted on the development of trust. *“An important role is for us to bring these clients in to clinic, and that’s their first contact and they can develop trust about coming to clinic and then they’ll develop trust about taking their child to school. So necessarily keeping them with services in the home I don’t think is always in their best interest”* (FG4 CaFHNs). Once a family trusts a service, they promote by telling others. “It’ll take a while, but the word will get out there eventually, the word gets out there.” (FG4 CaFHNs).

#### The importance of relationships

There was consensus among health professionals that developing respectful personal relationships with families is important and overarches continuity of care. *“The rapport established with the Nurse, that determines a lot of whether the client continued to want to engage”* (FG6 CaFHNs). The level of family engagement with services may therefore depend on the individual skill sets of health professionals. It is somewhat easier for Aboriginal workers to interact with Aboriginal families. For example, they may have insights into the family. *“The ACCFSCs might really know the history of the family quite well”* (FG7 CaFHNs).

Non-Aboriginal professionals working in mainstream services desired to build relationships with Aboriginal clients, but this did not always happen. *“They’re [CaFHS] very nosey and not always, they don’t always build rapport before they start asking questions”* (FG3 ACCFSCs). This posed a dilemma regarding how to make these relationships happen when what works for some families does not work for others. Furthermore, a ‘tick the box’ approach to care was seen as depersonalising and tokenistic. *“There's never any time for that rapport building. You try and leave it in there throughout your visit, but you also have forms that you have to complete and health checks you need to be doing”* (FG5 CaFHNs).

Some services for Aboriginal families are provided by a mainstream professional working in conjunction with Aboriginal counterparts. In these circumstances, it was questioned who decides and who does what. When working in a team, delineation and relationships between colleagues are important. *“It depends on who the ACCFSCs is and your relationship with them”* (FG7 CaFHNs). Interservice procedures are not always well documented or known, therefore linkages fall back onto individuals and individual relationships. When these relationships are not present interservice referrals collapse and decisions are reliant on individual health worker preferences.

Relationships to community are important to Aboriginal families. Non-Aboriginal workers often lacked connections to community yet realised these connections may not be appropriate. One Nurse said *“We don't often see Aboriginal clients in our Getting to Know Your Baby groups. And that's a shame”,* whilst another said, *“It would be nice if they actually had Aboriginal families together [with an Aboriginal worker]”* (FG6 CaFHNs).

Ideally, relationships with services would begin before there was a need for families to use them. *“It’s especially important with our cultural groups, if they get to know you before they actually need your service”* (FG7 CaFHNs). Perceptions of services are important. For example, families may be unclear about the role of workers. *“It took a long time for them to warm to us I suppose because they thought we were on the side of welfare”* (FG1 CaFHNs).

### Main theme 3 “We’ve got to be allowed to do it in a different way”

#### The system is racist

Facilitators to cultural safety for Aboriginal families woven into mainstream services included the employment of some Aboriginal staff, asking the family if they would like an Aboriginal worker or service to be involved, actively seeking understanding of the family’s cultural genogram and kinship ties, and providing non-Aboriginal staff with cultural awareness training. Nurses asked the family about who was important to baby. *“We’ve got an ‘our families, our support’ form. So, it says who lived in your house and who are the people that are important to baby rather than who’s your family, because they might not talk to any of them”* (FG7 CaFHNs). Whilst in place at policy level, the above recommendations were not always enacted in culturally safe ways. It was pointed out that culturally safe organisations worked with community appointed Aboriginal consultants, provided equitable services, had living, and working cultural safety documents and promoted interagency liaison with the Aboriginal community.

Use of a deficit model was evident when attributing care for Aboriginal families *“The thing is because we’re looking after Aboriginal families, not every family is high risk so it’s an assumption, like, there’s assumptions embedded in there. That’s your institutional racism”* (FG2). Further, culturally safe practices were not always evident. There were reports of cultural safety for Aboriginal families being compromised for example when non-Aboriginal staff were called upon to backfill Aboriginal workers due to staff shortages. Western ways of working took priority as discussed in FG 3: *“We’re still governed by a hospital and the hospital is a business and it’s a white business …a Western business. We [Aboriginal people] like to do things differently”* (FG3 ACCFSCs). Furthermore, non-Aboriginal health professionals identified needing additional support to be able to provide cultural care and at times appeared unwilling to share power. *“If it’s continuity of care around Aboriginal families with the mainstream health then we need Aboriginal people to be walking alongside us”* (FG7 CaFHNs). While this appears helpful, with an Aboriginal family the call would be for the mainstream service to be walking alongside the Aboriginal families. Such experiences lead to organisations having a poor reputation amongst community. *“There’s a lot of women in community that don’t like CaFHS because CaFHS are quite invasive when they go into the homes”* (FG3 ACCFSCs).

Culturally safe care was compromised when delivered by Nurses who identified as not being knowledgeable of Aboriginal ways. For example, a child and family health Nurse in FG 4 said, *“I hadn't taken out the specific Aboriginal support information. And… I don’t feel comfortable to [work with Aboriginal clients] because I'm not really versed in what exactly Aboriginal groups are”* (FG4). FG 1 stated outright that amongst non-Aboriginal Nurses *“there is still racism among some”* (FG1 CaFHNs). On the other hand, some non-Aboriginal Nurses were acutely aware of being from a white European female background and identified the impact this could have when working with Aboriginal families. *“I'm a white European woman walking into their… culture, I don't fit in that culture, there needs to be more [training]”* (FG6 CaFHNs).

Participants spoke of professional development learning opportunities such as cultural training, and policies related to cultural safety. They also spoke of Aboriginal Cultural Child and Family Support Consultants being engaged to work with leadership. This was all seen as insufficient. *“It’s not enough just to give us the cultural training, we need them [AMIC workers and ACCFSCs] as colleagues”* (FG7 CaFHNs). A non-Aboriginal Nurse suggested that cultural awareness training be Aboriginal led. *“It’s up to the Aboriginal staff to say, this is what we think that you need to know, that you need to understand so that you are respecting and honouring and working the right way”* (FG7 CaFHNs).

Workplaces are not always experienced as culturally safe for Aboriginal workers, with experiences of conflict of interest and disrespect. A participant in FG 6 explained that *“sometimes there's also a conflict of interest for Aboriginal workers in the community … And so that stress, they take that stress home because they know that person and know they're seeing it professionally and then, so it overlaps and that's a very big problem”* (FG6 CaFHNs). This is exacerbated by the ongoing lack of Aboriginal staff. Further the involvement of Aboriginal consultants was not well respected and their presence tokenistic. *“ACCFSCs would benefit from … being more respected and having a voice and doing it all and not sitting there just as…[someone] who just comes along because the family’s black. They need to have more of an active role”* (FG3 ACCFSCs).

#### Call for decolonising the system

The system influences all aspects of continuity of care including approaches to and provision of heath care, values, and relationships. Yet the system is not what health professionals want it to be for Aboriginal families. *“We’ve got to be allowed to do it in a different way”* (FG6 CaFHNs). They resoundingly argued for a system that focuses on values with *“cultural safety at the centre”* (FG6 CaFHNs). A whole system change was suggested to overcome hierarchical and political barriers to integrating services. *“The problem with what we’ve got at the moment is it’s still sitting within mainstream and it’s still influenced, I won’t use the word dictated, but it’s still influenced…and it’s controlled by things that are outside our control as Aboriginal people”* (FG3 ACCFSCs). Similarly, *“It’s almost sometimes like a them-and-us kind of… We’re not, we’re all together but it just doesn’t communicate like that”* (FG2 Midwives). Mainstream systems were not provided in an “Aboriginal way”.

Continuity of care is impacted when services do not work well together or support each other. While there are informal links between Aboriginal and non-Aboriginal services, often these are not formalised. *“It needs to be formalised, it needs to be a procedure, yes, and it needs… People need to be trained … whose services are involved? Ring those services and bring us together and work with us, not just this *ad hoc* ringing and *ad hoc* emailing, that’s how families fall through the gaps”* (FG4 CaFHNs). Formalising these relationships and links requires mainstream services to recognise the value and place of Aboriginal health services for families, rather than seeing them as an add on to mainstream service.

In addition, Aboriginal and non-Aboriginal services were reported to not communicate well. *“We don’t communicate, and we’re doing a lot of the same stuff”* (FG4 CaFHNs). An Aboriginal worker not attending a pre-arranged visit with a non-Aboriginal worker is another example of this. *“I found it really frustrating because one the worker wasn't there, two, the worker can't really tell me what support she has or hasn't got. So, I don't actually know what supports I should be trying to put into place”* (FG8 CaFHNs). The links that Aboriginal services have with child protection services are also problematic. *“Certainly, with child protection it's so important that we work together with the Aboriginal Cultural Child and Family Support Consultants too. There's in the past there's been issues with that, I think. Often, they're inaccessible like they're off sick or whatever. There's no good support for us when we're worried about a child's safety”* (FG6 CaFHNs). Without strong interservice relationships service continuity collapses.

#### Ways forward

There were many suggestions for a ‘utopian’ or ideal model of care which was Aboriginal led, valued Aboriginal ways of knowing and being, and designed with continuity of care and constant follow up principles in mind. *“To be gold standard I think it does need to be Aboriginal led, Aboriginal designed, Aboriginal staff”* (FG3 ACCFSCs). Furthermore, *“A gold standard to me, which we don’t have enough of, is if we had Aboriginal practitioners that actually did the work”* (FG7 CaFHNs). Such a service would be promoted before pregnancy and take time to build rapport with families. *“It’s especially important with our cultural groups, if they get to know you before they actually need your service”* (FG7 CaFHNs). The service would be accessible and safe, with clinics and outreach available. Roles within existing services may be revised to accommodate this. *“I think Child and Family Health maybe need to look at the Aboriginal Health Practitioner role and how they can specialise and tailor it towards the Child and Family Health work that they do”* (FG1 CaFHNs).

Overall, practical suggestions for professional development included avenues for exchange opportunities to work remotely and receiving cultural training led by Aboriginal staff. The participants called for a whole of systems change, especially systems and pathway designs that supports service engagement, such as working in a preventative manner with an antenatal start.

The health professionals highlighted a practical application that embed past learnings, allow for flexibility. Many commented that roles need focus and that the system needs more ACCFSCs, AMIC workers and Aboriginal Nurses and Midwives. Ultimately connecting to community and other services was seen as key to success in working with and providing continuity of care for Aboriginal families in mainstream health.

## Discussion

This study explored how continuity of care in the first 1000 days was perceived by AMIC workers, ACCFSCs, CaFHNs, and Midwives working in mainstream health services in South Australia. The three key themes emerging from the data were the system takes priority, culture is not central in approaches to care, and ‘we’ve got to be allowed to do it in a different way’.

We were told of a mainstream health care system where continuity of care for Aboriginal families across the first 1000 days is delivered inconsistently and often in culturally unsafe ways. Inconsistency and limitations were reported by health professionals to be the result of differing internal services received by Aboriginal families, challenging relationships between internal service divisions, a workforce who have differing capacity to deliver care in culturally safe ways, a wide range of approaches to care delivery, and ultimately inconsistency in relationships between care providers and recipients of care.

Canadian research from other Indigenous population groups have found that targeted, low-barrier bridging services to engage interdisciplinary interventions can promote continuity of care by offering timely and responsive service provision, including timely connection to long-term services and supports, appropriate individualised services and effective co-ordination of services [48]. Leaving families with complex support needs to be their own health navigators journeying through the multitude of services available is currently not working for Aboriginal families in metropolitan and segmented health care systems. There is no consensus on navigation roles and models in primary care. This study shows that health care staff perceive that Aboriginal families experience fragmentation and gaps in service delivery. Perhaps innovation and systems navigation provided by individuals or teams is a solution to overcome the barriers, as suggested by research from the US identifying health navigation as an emerging strategy to reduce barriers to care [49].

On an individual level some health workers described trying to develop relationships that facilitated culturally safe care with Aboriginal infants and their families. They told of being thwarted by required approaches to care, the limitations of cultural capability of some care providers, and limitations on the relationships and communication systems within and between internal service divisions. Overall, where culturally safe practice was attempted, it was often not supported by the system. Culturally safe practice appeared as an appendix to mainstream care, rather than an embedded and valued approach to care. The system did not appear to change to become more culturally safe, it merely shifted slightly to accommodate difference, rather than embracing and embedding Aboriginal ways of knowing and being. For example, the Aboriginal Family Birthing Program is an award-winning program within mainstream health services, providing culturally safe care to birthing women and their families [50]. Despite this, health workers report that families are challenged to move between this program and Aboriginal Community Controlled Services and often experience culturally unsafe care and racism when they are required to shift across to main-stream services.

The system was reported as one that was disjointed within mainstream and between external services across the first 1000 days. This disjuncture represents a core of system racism. Manifestation of systemic racism is an inequitable system of practices and structure that contributes to exclusion, is rooted in past and ongoing colonisation of which health care systems are a part of with biased behaviour, inequitable practices, and in which racist attitudes and comments are sometimes tolerated [51, 52]. This is supported by other research finding racism a serious ethical issue across health sectors [53]. Racism is present in society's systems and ingrained in social structures and institutions [54]. Henricks [54] identifies that prejudice, bias and poor attitudes are some aspects of racism, but that pervasive institutional racism occurs regardless of individuals’ or groups’ good intentions. Racism is a significant social predictor of health, and numerous studies shows evidence of racial attitudes, feelings, or actions among healthcare workers, both implicit and explicit [55, 56], resulting in “lower levels of healthcare-related trust, contentment, and communication” in the healthcare system ([57], p. 1). Minority health outcomes are worsened by the combination of poor health care services and underutilisation of healthcare [58]. Health care system deciders must understand that cultural safety in a system is built through time, not only in a one-time "check box" session. It requires gradual, inclusive, comprehensive, and systematic change.

Main-stream health care systems are large and have limited capacity to be nimble. Despite this, it is not acceptable for cultural care to be relegated to the periphery, with culturally safe practice as an appended tick box activity. We suggest turning the tables so that culture is centralised across systems, services, and service provision. The current drivers of care do little to centralise cultural care. For example, current discourses of care include patient centred and family centred care [59]. This approach is not appropriate for Aboriginal families because a care model needs to include community. Individuals and families are intrinsically part of community and culture [60]; we need to implement culture centred care, similar to the Aotearoa model that centralises Māori culture in care [61]. Aboriginal and Torres Strait Islander ways of knowing and being, need to be at the middle of the system rather than at the periphery. Placing culture at the centre will support all peoples in the ‘mainstream’ not only Aboriginal and Torres Strait Islander peoples, as it speaks to the importance of values-based care.

Participants spoke of the need to approach care from a position of values, specifically trust. Fear and distrust of mainstream health services due to historic colonisation is real. Health care systems that do not understand the legacy of colonisation may seem surprised that families will not return to use their services but should not be surprised. Trust requires mutual respect. Systemic and individual racism is an indicator of the absence of respect. Both of these were identified and described by health care workers. Without trust nothing will change. Using the cultural safety principles ([41], p. 15) as a guide, during the whole process we asked participants to reflect on their practice and provision of culturally safe care across the continuum and to consider their interactions with Aboriginal clients for and with whom they cared. We then looked at where power differentials existed. Although many health care professionals felt powerless in the system, some participants focused on minimising power differentials by focusing on the developments of relationships in spite of system pressures. This is enactment of cultural safety principles, such as reflecting on your own practice, minimising power differentials between yourself and clients, engaging in conversations with clients, treating people regardful of their cultural or individual differences, and undertaking a process of decolonisation ([41], p. 15). Can we see a process of decolonisation in data in this research? The only place decolonisation is seen is at individual level where health care professionals are working around the edges providing culturally safe care and prioritising building relationships. Certainly, the system is not providing decolonisation of services or health care—however a system who prioritise relationships enable culturally safe continuity of care.

Provision of culturally safe care must be taken seriously by services and systems—not just as something health workers have to tick off when attending an annual workshop. Aboriginal staff could work with non-Aboriginal health care staff to help them develop insights into practices and realise the impact of own practices. Additionally, health workers must develop insights into the systems and structures within which they work and not just focus one’s own patch. Health workers have limited capacity to make change on a system level, but can act as change agents from within, and at the margins, albeit not at the top.

### Strengths and limitations

Strengths of this research is the broad engagement with both Aboriginal and non-Aboriginal health care professionals as experts in service provision to Aboriginal families to identify and implement patient and culture-centred strategies to improve access and acceptability of care. Their perspectives for family care are particularly important when working with other than majority populations.

Despite the strengths, there were some limitations in this study. Most health care professionals who participated worked in an urban area; therefore, the perspectives of staff working in regional or remote areas were not identified. Every Aboriginal community is unique, the perspectives shared are not intended to be representative or applicable to all communities or health care settings working with Aboriginal families in Australia or otherwise.

#### Implications for health policy and systems

- Cultural safety must be taken seriously by health systems and services—not just ticking a box when attended a workshop on
- Health workers need to develop insights into own practice and the impact of own practice
- Health workers must develop insights into the systems and structures within which one works and not just focus on one’s own patch
- Recommendations for improved culturally safe practice across the continuum and improved models of care

## Conclusion

This research highlights a lack of continuity of care for Aboriginal families accessing mainstream health services from the antenatal period through to an infants’ first 1000 days of life. This research has implications for communities, and it calls for strategies to enhance continuity, and healthcare services to provide appropriate and culturally safe care.

The participants in this study called for a mainstream health care system with cultural safety principles embedded at the core, rather than appearing as an appendix and an afterthought. Adding cultural bits and pieces to models of care does not make a culturally safe system that invites Aboriginal families into a partnership. What we have is a system that overarches individual relationships and services, but participants want Aboriginal community and family values to form the system not the opposite way around.

This research explored workforce perspectives of culturally safe care experiences and satisfaction with care provided to Aboriginal women and their infants in mainstream health. The findings will inform and guide future changes to improve continuity of care for Aboriginal families and workforce in health policy, systems and services, and to inform culturally safe care that meets best practice, and enables families’ access to mainstream health services.

Findings will inform and guide future changes to improve continuity of care for Aboriginal families and infants in the first 1000 days.

## Supplementary Information

**Additional file 1.** Interview guide.

## Data Availability

The datasets used and/or analysed during the current study are available from the corresponding author on reasonable request.

## Abbreviations

ACCFSCs: Aboriginal cultural child and family support consultants
AMIC: Aboriginal maternal infant care [worker]
CaFHNs: Child and family health nurses
CaFHS: Child and family health services
