# Supplementary material for: A call for culture-centred care: exploring health workers' perspectives of positive care experiences and culturally responsive care provision to Aboriginal women and their infants in mainstream health in South Australia
Source: Health Res Policy Syst. 2022 Dec 12;20:132. doi: 10.1186/s12961-022-00936-w (PMC9743671; doi:10.1186/s12961-022-00936-w)
Supplement: Supplementary file 1 — Additional file 1. Interview guide. [file 12961_2022_936_MOESM1_ESM.docx]

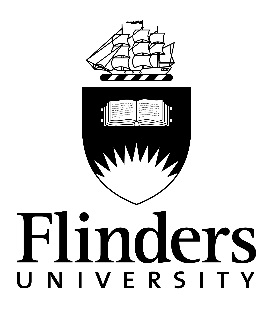


**Interview Guide**

| *Building nursing and midwifery capacity to maintain continuity of care for Aboriginal families and children accessing mainstream health services in the first 1000 days* |
| --- |

**Health Care Professionals**

Thank you for having this interview (individual or group interview pending on the participants’ choice) with us. What we want to talk about today is how important it is for Aboriginal and Torres Strait Islander families to get a service that is continuous from when baby is born through to when baby is 2 years old.

- Can you tell us about how continuity of care works or operates for Aboriginal families in your care?
- Can you tell us about how you enable continuity of care for the families you work with?

Prompt-what makes this easier or difficult?

- In your opinion, how is this working for you and the women that you care for?
- Can you tell us about how you manage transition in care between services?

Prompt-what works well and what could be done better?

- What consideration is given to maintain the cultural safety of the Aboriginal families in your care?

Prompt-what works well and what could be done better?

- How would you like to see a model of continued care for Aboriginal families working?
- What needs to change to make this happen?
- Is there anything else you would like to share with us?
